# The Complete Works of Hippocrates, a New Translation, with the Greek Text on the Opposite Pages, Collated with the Manuscripts and All the Editions; with an Introduction, Medical Commentaries, Various Readings, and Philological Notes; Followed by a General Index of the Matter Therein Contained

**Published:** 1844-04

**Authors:** 


### ART. XI.

1. *Œuvres Complètes d'Hippocrate, Traduction Nouvelle, avec le Texte Grec en regard, collationné sur les Manuscrits et toutes les Editions; accompagnée d'une Introduction, de Commentaires Médicaux, de Variantes, et de Notes Philologiques; suivie d'une Table générale des Matières.* Par E. LITTRÉ, Membre de l'Institut et de la Société d'Histoire Naturelle de Halle.—*Paris*, 8vo, vol. I, 1839, pp. xvi, 637; vol. II, 1840, pp. lv, 717; vol. III, 1841, pp. xlvi, 563.

*The Complete Works of Hippocrates, a new Translation, with the Greek Text on the opposite Pages, collated with the Manuscripts and all the Editions; with an Introduction, Medical Commentaries, various Readings, and Philological Notes; followed by a general Index of the matter therein contained.* By E. LITTRÉ, &c.

2. *Hippocratis Nomine quæ circumferuntur Scripta ad Temporis Rationes disposuit* CHRISTIANUS PETERSEN, in Gymnasio Hamburgensium Academico Philol. Class. Prof. P.; pars prior. *Præmissum Indici Lectionum in Gymnasio Academico A.* 1839 *habendarum.—Hamburgi*, 1839. 4to, pp. viii, 55.

*The Writings which commonly go under the Name of Hippocrates, chronologically disposed by* CHRISTIAN PETERSEN, &c.

"WHAT from its antiquity," says James Harris, in the preface to his *Hermes*,[a] "is but little known, has from that very circumstance the recommendation of novelty; so that here, as in other instances, extremes may be said to meet:"—and accordingly we think that there may perhaps be more of the interest of novelty in occasionally laying before our readers a few sketches of the origin and early history of different branches of medical science, than in giving an account of the latest discovery or the newest theory. We must confess too that our editorial conscience has been somewhat troubled from time to time at the thought of our having so sparingly fulfilled our early promise of now and then making room for retrospective reviews of works, which once enjoyed a just celebrity, and which are still worthy of being remembered; and therefore we were not sorry to have an opportunity offered us on two recent occasions of partially redeeming our credit with our more learned readers both at home and abroad, by saying a few words on the anatomy and physiology of the ancients, and thus proving that even in these branches of science (which are universally agreed to be one of their weakest points), our fathers were not quite so ignorant as some of their undutiful children are apt to think them. And still more gladly have we hailed the appearance of M. Littré's work, both because we think it seems likely to supply in a great measure a desideratum in medical classical literature, which all competent judges have long acknowledged, and which several of the most learned members of our profession have

[a] [The writer has begged us to say that this sentence was written before the appearance of our last April Number, in which he found that the same quotation (though taken from a different work by the same author), was made use of in a similar manner at the close of the review of Dr. Greenhill's Theophilus.—ED.]

in vain endeavoured to fill up,[b] and also because it forces us in a manner to discharge a duty which might otherwise have been driven off for an indefinite time, and to offer some account of the life and writings of one of the oldest and ablest of the ancient physicians, "the divine old man," "the father of medicine" himself.

It is certainly no affected humility, but the very simple truth, to acknowledge that we enter upon this subject with no ordinary feelings of diffidence and distrust; for we cannot but call to mind the names of some of those great men who during a period of more than two thousand years have commented on the writings of Hippocrates, and have employed their utmost critical acumen in the endeavour to separate the chaff from the wheat, and to determine which among the numerous treatises that bear his name have really a right to claim him as their author. In the present instance, however, the labours and difficulties of our task have been much lightened by the works before us, especially that of M. Littré, which is at once so complete, so learned, and so accurate, that we think we cannot do better than lay before our readers a tolerably full abstract of his Introduction, making such references to the two other works as we may from time to time feel necessary, inasmuch as they do not seem to us to require so careful a perusal nor so complete an analysis. We must also reserve to ourselves the right of occasionally differing from each of the learned writers; and, finally, we trust they will not think us anxious to depreciate the merit of their works if we supply a few omissions, and correct one or two mistakes,

"quas aut incuria fudit,
Aut humana parum cavit natura."[c]

[b] Of these it may be sufficient here to notice particularly two, the latest and the most valued: 1st, Adamantius Coray or *Koray*, (born at Smyrna, 1748, died at Paris, 1833,) who published a learned and excellent edition of Hippocrates 'De Aëre, Aquis et Locis,' in Greek and French, 2 vols. 8vo, Paris, 1800, and whose name was well known in the literary world as the editor of several other classical works; and 2d, F. R. Dietz, who died at Königsberg in 1836, at the early age of thirty-seven, and who had spent several years in travelling almost all over Europe partly at the expense of the Prussian government, for the purpose of collating Greek, Latin, Arabic, and Sanscrit medical manuscripts. The principal fruit of his labours, which he lived to publish, is to be found in his 'Scholia in Hippocratem et Galenum,' 2 vols. 8vo, Regim. Pruss. 1834, which are interesting and valuable, though bearing evident marks of carelessness and haste; but the greater part of the remainder of his manuscripts is being now edited by J. L. Ideler at Berlin, of which two volumes have already appeared under the title 'Physici et Medici Græci Minores.' [Since writing the above sentence we regret to say that we have been informed of M. Ideler's premature death, an event which must be a source of much regret to all who take an interest in ancient Medical Literature. We have not heard who has been appointed by the Prussian government to continue the work.]

[c] In performing this last part of our duty, (which we would always gladly be spared, though some of our ill-natured readers believe that it is precisely that in which we take most pleasure,) we are saved a good deal of trouble by the fact of our not having noticed the works earlier; the consequence of this is, that almost all M. Littré's oversights have been already discovered and pointed out in other journals, and we think it would not be consistent with our duty either to the author, to our readers, or to ourselves, to bring forward a long list of errata, which he has himself freely acknowledged and corrected in the second and third volumes of his work. The critiques that have come to our knowledge are: 1, in Miller's 'Revue de Bibliographie Analytique,' tome i, pp. 219, 506, tome ii, p. 892; 2, by Dr. Ermerins, in the 'Hallische Allgemeine Literatur-Zeitung,' 1839, Oct., pp. 179-80; and 3, by M. Rosenbaum, in the 'Archiv für die gesammte Medicin, herausgegeben von Dr. H. Haeser,' B. i, Heft i.

We could have wished to have given some account of the labours of M. Littré's predecessors, but as this would take up too much space, we must refer our readers to the first volume of his work, where (pp. 540-54,) he will find a full and candid estimation of all the previous editions of the complete works of Hippocrates. Before, however, proceeding to analyse the contents of the work, we cannot refrain from saying a few words on its external appearance and arrangement; and this we shall do with the less scruple, in the hope that the editors employed on the ancient physicians by the Sydenham Society, may be able to extract from our remarks some few hints that may be useful to themselves.

The most prominent external defect is the omitting to mark in the margin of each page the corresponding pagination of the principal former editions, which is of the more consequence, as the treatises are arranged in quite a different order from that which has usually hitherto been followed; and the result of which is, that any attempt to find a passage quoted by the pages of any of the common editions (such as that of Foes, Vander Linden, Kühn, &c.) is almost hopeless. This omission alone is quite sufficient to render the work so incomplete as entirely to prevent its ever superseding the use of Kühn's edition, of which the insertion of the corresponding pagination of Foes, Chartier, and Vander Linden, is almost the only excellence, but which, with all its imperfections,[d] seems still likely to maintain its place as the most popular edition, until we obtain a complete and uniform collection of the works of the principal ancient physicians, equally commodious in size, type, &c., and of greater critical value.[e]

Another defect, in our opinion, is the mixing together at the bottom of the page the various readings, the shorter notes on the text itself, and the longer annotations referring to the subject-matter; whereas it would have added no less to the beauty of the book than to the convenience of the reader, if these last had been placed together at the end of the volume.

[d] The critical merits of Kühn's collection (except perhaps the Aretæus and Dioscorides) are certainly very small, and yet we must acknowledge that it has done good service to the cause of ancient medical literature, first by furnishing us with an edition of these old writers in a less cumbrous form than the huge folios of the sixteenth and seventeenth centuries; and secondly, by exciting an interest in the subject which thirty or forty years ago was almost extinct, and which has been continually increasing from that time to the present. It ought also to prevent our speaking too harshly of Kühn's editorial labours, if we remember that at the time of the appearance of the first volume of his Collection (1821), he was already in his sixty-seventh year, a time of life when few persons would have the courage to undertake so vast a work.

[e] A work such as that which we have been contemplating is far too great for any single editor to attempt, (as, indeed, the example of Kühn may prove,) nor (now that the days of Aldus, and Stephens, and Elzevir are gone by,) would it be easy to find a publisher who would be willing to bear the risk and expense of such an undertaking in the cause of medical literature—unless, indeed, it should happen to be comprehended in one of the magnificent schemes of M. Didot. However, it is to some medical college or association, like the Sydenham Society, that we have a right to look for the accomplishment of this project in a manner worthy of the country of Linacre, Caius, Freind, and Wigan; nor do we think so meanly of the classical taste and learning of our medical brethren in Great Britain as to doubt that, when once their attention has been called to the subject, some few fit and proper persons may be found able and willing to act as editors.

A short marginal analysis of the contents of each chapter would have been a great assistance to the reader in finding any particular passage. This is found in several of the best modern editions of Greek and Latin historical works; and though the example has not hitherto been frequently followed by the editors of the ancient physicians, we hope that the plan may be more generally adopted for the future, especially as it will give very little additional trouble to the editor.

Before we finish these few introductory observations, we cannot help remarking the very great prolixity of the general Introduction, of several of the Arguments prefixed to each treatise, and of some of the notes, which might very easily have been compressed into little more than half their present length. On this subject we cannot do better than quote a few lines from a critique in the 'Quarterly Review,' (vol. 64, p. 378,) on the Æschylus of Mr. Peile, who had fallen into the same error, which, indeed, few writers of notes in the vernacular tongue have managed to avoid. "This comes of the 'fatal facility' of *French* note-writing! *French* is as unfit for notes, as Latin is for Lexicography. Latin is in itself the language for notes; and there are, besides, extrinsic advantages: Latin notes, for instance, must be terse; here is one check to prolixity; Latin notes cost most men a good deal of trouble; here is another." And though we are not quite sure that we should be inclined to extend this dictum to biographical and historical Introductions in general, yet in the present instance we confess we would rather have had to read about three hundred pages of Latin, than *five hundred and fifty-four* pages of French.

We might mention several little particulars in which we think the beauty of the work might have been increased, but we must not forget that "*beauty*" is a vague and arbitrary term, nor attempt to force a foreigner to adopt our English notions on the subject of taste. We therefore gladly proceed to give some account of the internal and more important merits of the work.[f]

M. Littré tells us in his preface, (p. vii,) that his labours on the works of Hippocrates have had a three-fold object, viz., the revision of the text, the furnishing a correct translation, and a medical commentary. The first part of this task he found much more troublesome and laborious than he had expected, as the text of the writings of Hippocrates, (or, as M. Littré calls them for brevity, "the Hippocratic Collection,") had received very partial correction since the time of Foes, and remained almost in the state in which it had been left by him. In order to remedy this defect, M. Littré has collated, (and apparently with great care,) all

[f] We may, however, notice one very singular peculiarity, which must have been more cumbrous to the editor himself and to the printer than to the reader; we allude to his plan of designating the different manuscripts in his notes, not by certain conventional letters, (A, B, C, D, &c.) as is usually done, but by their actual numbers in the catalogue of the Royal Library at Paris, viz. 2140, 2142, 2253, &c. The consequence is that he has used at least three times as much space as was necessary, and *that*, too, without any conceivable advantage. However, the editor himself seems to have been fully sensible at last of the clumsiness and inconvenience of this method of quoting the manuscripts, (we only wonder how he could ever have thought of it!) and accordingly he has in the latter half of the second volume adopted the shorter and more usual one. (See tome ii, p. 378.)

the MSS. of the Royal Library of Paris, to the number of sixty-two,[g] (tome i, p. 511, &c.,) from which he has derived the most valuable assistance. The various readings that he has collected are placed at the bottom of the page, and the reading that he has adopted in the text is discussed and explained with greater or less detail as may be required. He generally follows the manuscript readings, but in certain cases he has departed from them, (p. viii,) in conformity with the rules respecting the use of the Ionic dialect in the Hippocratic Collection, which he has laid down and fully explained in an appendix. (p. 479, &c.) He tells us that it has been his aim to place these writings completely within the reach of the medical practitioners of the present day, so that they may be read like so many contemporary works. (p. ix.) In prosecuting this design, however, he found two great difficulties present themselves: the first arising from the ancient medical theories, which have long ceased to be familiar to the modern practitioners, and which, nevertheless, must be known and understood by any one who wishes to enter into the full meaning of the ancient medical writers; the other difficulty was occasioned by the fact that these works are written in a dead language, in which the medical terms have sometimes a doubtful signification, and sometimes even a deceptive one, inasmuch as the meaning which they bear in modern phraseology is not unfrequently quite different from that which they bore in the writings of the ancients.[h] In order to obviate the former difficulty, M. Littré has given not only a general Introduction to the whole Hippocratic Collection, but also a preliminary Argument at the head of each separate treatise, containing all the information that seemed to be necessary for the due understanding of the work; and with reference to the latter, he has endeavoured to express the meaning of the ancient technical terms as precisely and definitely as possible, an attempt which (as he says himself,) often forced him to undertake a retrospective diagnosis, attended with no less obscurity than that at the bed-side. (p. x.) He quotes some remarks by Grimm, (p. x,) on the use of vernacular translations, with which we fully agree, as there are probably many members of the profession who would be glad to become acquainted with the works of the ancient physicians, but who are unable to read them with ease in the original language or in a Latin version; we think, however, that vernacular translations are likely to be much more useful in every respect if published in a separate form, than if appended to the original text and the necessary critical notes, &c., which of course more than double the price of the work, and (in the eyes of the English reader,) add little or nothing to its value. M. Littré apologises for the length of his introduction, (pp. xi, xii,) which certainly might have been written in a less diffuse style without any detriment to the work, and greatly to the comfort of the reader; and he then proceeds to mention some of the principal alterations that he intends to make in the arrangement of the works composing the Hippocratic Collection, (pp. xii, xiii,) some of which he is going to omit entirely, as being merely a repetition

[g] Of this large number, only *two* contain the whole of the Hippocratic Collection, most of the others having only one or two of the smaller and less important treatises.

[h] This latter source of obscurity has been noticed and illustrated by a few examples in an article in our Twenty-ninth Number on Prof. Marx's 'Herophilus,' p. 107.

of what is found in another place,[1] and one of which, (viz. the treatise *De Septimanis,*) he will publish for the first time in an old Latin translation that he has discovered in the library at Paris.

In the first volume, which consists of six hundred and thirty-seven pages, only a single work of the Hippocratic Collection is contained, viz. that entitled Περὶ Ἀρχαίης Ἰατρικῆς, *De Veteri Medicina,* which extends from p. 555 to the end; the other five hundred and fifty-four pages being occupied by the Introduction, which treats of various matters connected with Hippocrates, and contains, indeed, almost everything that need be said on the subject. But his object in this Introduction shall be stated in his own words, (pp. i, ii:)

"Do the medical works which have come down to us under the name of Hippocrates, all really belong to this physician? If not, who is the author, or who are the authors, whose works have been preserved under a false name in the Hippocratic Collection? By what mark may we distinguish the writings which really belong to Hippocrates from those which do not? What classification must we introduce into this mass of works, if we succeed in proving that they are derived from different sources? How has it happened that writings have been falsely attributed to Hippocrates, and have been published under his name? To what epoch can we trace back the publication of this celebrated Collection? Was it published during the life-time of Hippocrates himself, or was it not given to the world in its present form till a considerable time after his death? What is the true system of this physician, when we have put aside the books which do not belong to him? In what way was his system attached to the more ancient doctrines, and what immediate fruits did it produce? Lastly, what do we know with certainty about the life of Hippocrates himself, in the midst of all the fables with which it has been embellished? And what certain notions can we form to ourselves of his system, of his way of looking at things, and of his medical character? These are the questions, (each of which contains in itself several others,) that I propose to discuss in the long essay to which I have given the name of *Introduction,* and which I here submit to the judgment of the reader. The further I have advanced in the translation of the Hippocratic Collection, the more clearly I have seen the necessity of carefully discussing all these questions. They are only preliminary, it is true; but they are not on that account the less essential; and amidst the difficulties of the new edition that I have undertaken, I have only felt some degree of certainty from the moment when I solved the problems of literary and medical criticism, which I have just enumerated."

Accordingly, after giving in the first chapter, (pp. 3-26,) a sketch of the medical science before the time of Hippocrates, he proceeds to give an account of the life of Hippocrates himself, (chap. ii, pp. 27-43,) and to describe generally the Hippocratic Collection, or works bearing his name, (chap. iii, pp. 44-65.) In the fourth chapter, (pp. 66-79,) he has collected all the passages relating to Hippocrates and his writings that are to be found in those authors who lived before the establishment of the medical school at Alexandria; in the fifth, (pp. 80-132,) he gives an account of the transmission of the Hippocratic Collection, and of the series of commentators in antiquity; and in the sixth, (pp. 133-53,) of the different lists that have been preserved of the works composing it.

[1] For instance, the eighth section of the 'Aphorisms,' which has always been considered spurious, and of which great part is to be found in the newly-discovered treatise 'De Septimanis;' and also the little works entitled 'De Diebus Criticis' and 'De Ossium Natura,' both of which are mere compilations, consisting of fragments taken from other parts of the Collection.

He then proceeds, (chap. vii, pp. 154-68,) to discuss the data or elements of criticism possessed by the ancients, by which they pronounced judgment on the genuineness of this or that work in the Hippocratic Collection, and to examine their value; and notices in the next chapter, (pp. 169-99,) the modern works which have professedly treated of this subject. This introduces (chap. ix, pp. 200-41,) a discussion on certain points of medical chronology, which tend to determine the genuineness or spuriousness of the different treatises. In the tenth chapter, (pp. 242-61,) he inquires into the relations which some of the works in the Hippocratic Collection bear to each other; and in the eleventh treats of the question of its formation and publication. (pp. 262-91.) After these preliminary matters have been fully discussed, he gives a critical account of each work separately, (chap. xii, pp. 292-439;) and lastly, we have an analysis, (chap. xiii, pp. 440-64,) of the medical doctrines of Hippocrates, and some remarks on his medical character and style. (chap. xiv, pp. 465-78.) In an appendix to the Introduction, he discusses the dialect in which the works forming the Hippocratic Collection are written, (§ i, pp. 479-502,) and gives an account of the text and editions published by the ancients, (§ ii, pp. 502-10,) of the MSS. contained in the Royal Library at Paris, (§ iii, pp. 511-39,) and of the complete editions and translations that have been published in modern times. (§ iv, pp. 540-54.)

Such are the contents of the Introduction, containing much matter for study suited both to the scholar and the physician; those parts, however, which are simply critical, philological, and bibliographical, (as being probably uninteresting to the greater number of our readers,) we shall notice no further than by saying that they seem to have been written with care and accuracy, equal to that which is displayed in the other portions of the work; of the remainder we shall now proceed to give an analysis, noticing chiefly (as far as possible,) those points which best suit the general character of our Journal, and adding occasionally a few observations of our own. To begin with the state of medical science before the time of Hippocrates, which forms the subject of the first chapter:

"When," says M. Littré, (p. 3,) "we search into the history of medicine and the commencement of the science, the first body of doctrine that we meet with is the collection of writings known under the name of the works of Hippocrates. The science mounts up directly to that origin, and there stops. Not that it had not been cultivated earlier, and had not given rise to even numerous productions; but all that had been done before the time of the Physician of Cos, has perished, and of these writings we have only remaining a few scattered and unconnected fragments. The works of Hippocrates have alone escaped destruction; and by a singular circumstance, there exists a great gap after them, as well as before them. The medical works from Hippocrates to the establishment of the school of Alexandria, and those of that school itself, are completely lost, with the exception of some quotations and passages preserved in the later writers; so that the writings of Hippocrates remain alone amongst the ruins of ancient medical literature."

In early times there were, according to M. Littré, (p. 5,) three sources from which the medical science of the Greeks was derived: 1, the different colleges of the priest-physicians, who served in the temples of Æsculapius, and took the name of Asclepiadæ,(Ἀσκληπιάδαι,) as being the descendants of the god; 2, the philosophers or physiologists, who employed themselves in the study of natural philosophy, including also the organization of bodies and the origin of diseases; and 3, the gymnasia, where the persons at the head of these establishments gave great attention to the effects produced on the health of the athletæ by different kinds of food and exercise.

M. Littré considers the worship of Æsculapius to have come to the Greeks from the east, (p. 6,) which is (to say the least) a very doubtful and disputed point;[j] but this mythological question does not concern us at present, nor need we give a list of the principal temples of the god, which soon became very numerous. They seem to have served not only for places of religious worship, but also for a kind of hospital for the sick,[k] and lastly, for a medical school for students. They would also appear to have combined, in some degree, the character of the modern "watering places," as they were built in pleasant and healthy situations, with a sacred grove at hand, (p. 10,) and, if possible, a mineral spring; thus offering to the visitors everything necessary both for health and amusement. The priests of the temple were the physicians; and the patients, when they went away, left behind them in gratitude to the god, a votive tablet, containing a short account of their disease, and of the means used for their recovery: these, in process of time, (as we shall see,) were turned to good account, and probably furnished the materials for at least two works in the Hippocratic Collection.[l]

The most famous of these medical schools before the time of Hippocrates were those of Cyrene, Rhodes, Cnidos, and Cos, (pp. 6, 7;) of these, the two former were soon eclipsed by the two others, which acquired a great reputation, and exercised an important influence on the art and science of medicine. (p. 7.) From the school of Cnidos proceeded the earliest work that can with any certainty be attributed to the Asclepiadæ, entitled *Cnidian Sentences*, (Κνίδιαι Γνῶμαι,) which is now lost, but which was extant at least as late as the time of Galen. (pp. 7, 8.) The Cnidian physicians appear to have divided diseases into a great number of minute species; for instance, they reckoned seven different disorders of

[j] Of the state of the medical art during the Trojan war, (or rather, probably, in Homer's own time,) which is the earliest period when we find a mere mortal exercising the healing art, the following sketch is given by Mitford, ('Hist. of Greece,' chap. ii, § 3, vol. i, p. 158.) "The knowledge of the cure of internal diseases made, it should seem, in Homer's age, no part of the science of physic. It is remarkable that the poet nowhere speaks in plain terms of sickness. Diseases, indeed, and mortal ones, are mentioned, but as the effect always of the immediate stroke of the Deity, and not of anything in the common course of nature. They seem thus to have been esteemed utterly beyond the reach of human skill to relieve. The epidemical sickness of the army before Troy was occasioned by the darts of Apollo, and could be removed only by the prayers of Chryses. That scanty knowledge of nature to which the age had arrived was applied only to relieve the effects of external violence upon the human frame. Skill in surgery was in the highest esteem; though it seems to have gone no further than to the extraction of the instrument of a wound, and the application of a few simples for stopping hæmorrhages, and assuaging inflammations. Charms and incantations therefore were sometimes called to its assistance, or even to supply its place. Ulysses, when very young, being wounded by a wild boar, the hæmorrhage was stopped by incantation."

[k] We believe that no mention of any establishment for the relief of the sick, more nearly answering to the modern hospitals than these temples of Æsculapius, is to be found among the ancients till after the introduction of Christianity. We know that some persons hold a contrary opinion, but we cannot here enter upon the proof of our own.

[l] Some specimens of these votive tablets, which are certainly very curious, may be found in Gruter's collection of Greek Inscriptions, or in the Histories of Medicine by Le Clerc and Sprengel.

the bile, twelve affections of the bladder, four of the kidneys, four species of strangury, three of tetanus, four of jaundice, and three of phthisis. (p. 8.) Those of Cos, on the other hand, paid more attention to the symptoms which announce the curative efforts of Nature, and to the distinguishing the different crises and the critical days. (p. 9.) They did not publish any works till after those of Cnidos, but the little treatise found in the Hippocratic Collection, and bearing the title of *Coan Prognostics,* (Κῳακαὶ Προγνώσεις,) is generally allowed to be anterior to the time of Hippocrates himself, and to be in fact a collection of medical notes selected by the Coan physicians from those deposited by the patients in the temple. (p. 9.) Of the great number of persons claiming to belong to the family of the Asclepiadæ, some few were really descended from Æsculapius, (as, for instance, the ancestors of Hippocrates,) but probably the rest were merely associated with them by initiation, (pp. 11, 12,) and it was perhaps on these occasions that the celebrated *Oath,* (which is placed at the head of the Hippocratic Collection,) was administered.

The results of the labours of the Asclepiadæ, (or priest-physicians,) before the time of Hippocrates, may be shortly summed up : medical attendance on sick persons both in and out of the temples; (or, in modern language, on the "In and Out-Patients of the Hospitals ;") an account on votive tablets of the chief peculiarities of each disease, and the mode of treatment; the collection of these notices; the publication of medical works; and the traces of two medical systems, one of which, (the Cnidian,) consisted in noting all the symptoms of a disease, and making of almost every one a distinct malady; the other, (the Coan,) inquired into what the symptoms had in common, as indicating the state of the patient's strength and the cause of the disease. (p. 13.)

The second source from which the medicine of the Greeks was derived, is to be found in the schools of the natural philosophers, several of whom composed works on physical science, of which nothing but a few fragments remain. (pp. 13, 14.) The most celebrated of these were Alcmaeon, Anaxagoras, Empedocles, Acron, Diogenes of Apollonia, and Democritus, whose most striking opinions M. Littré quotes, (pp. 14-21,) from which it appears that they dissected animals, inquired into the causes of disease, and endeavoured to introduce into the study of medicine doctrines corresponding to those which they admitted in their philosophical systems.[m] (p. 21.)

As the third great source of medical science, M. Littré mentions the gymnasia, (p. 22,) where the Greeks learned to reduce fractures and luxations, to examine into the kinds of food that were most conducive to health and strength, to observe what modifications of diet were necessary for different ages and constitutions, and to recognize the changes in outward appearance caused by any departure from the usual regimen. Besides this, they applied gymnastics to the treatment of diseases, and with such success, that many patients left the temples for the gymnasia, and the priest-physicians were induced to study the effects of these exercises, and admit them into their system of therapeutics. (p. 23.)

[m] There is an interesting little work on this subject, by Kühn, entitled 'De Philosophis ante Hippocratem Medicinæ Cultoribus,' reprinted in his 'Opuscula Academica Medica et Philologica,' Lips., 1827-8. 2 vols. 8vo.

From these three sources had sprung in the fifth century before Christ, a considerable mass of opinions and facts, derived from the study of disease in the Asclepeia, the study of health in the gymnasia, and the spirit of generalization in the works of the philosophers, (pp. 23-4 ;) and it is the union of these that forms the basis of the medical system developed by Hippocrates and his pupils. (p. 24.) For M. Littré is not diminishing the credit of Hippocrates, when he seeks to do away with the illusion entertained by many persons, that from his being called "the Father of Medicine," he was the sole and original inventor of medical science; whereas, it appears, that much had been done before his time, and that his chief merit consisted in developing, adding to, and forming into one complete and harmonious system, the results of the reasoning and experience of his predecessors. (pp. 24-5.)

The personal history of Hippocrates, which occupies the second chapter, need not detain us long, as all that is *known* of his life may be told in a few words, though to mention and refute all the absurd fables that have been invented concerning him would fill volumes. Besides, in a work like ours, which professes to be more medical than antiquarian, we fully agree with M. Littré, (p. 28,) in thinking that his literary history is of more consequence than his biography, properly so called; and that it concerns us more to know what he wrote, than what he did; the books which he composed, than the details of his daily life.

Hippocrates was born, not in Greece proper, but in Cos, a small island off the coast of Caria, probably in the year 460 before Christ. (p. 34.) He accordingly lived in the age of Pericles, and was the contemporary of Herodotus, Thucydides, Socrates, Democritus, Plato, and others of the most celebrated characters of antiquity. He was descended from Æsculapius by his father's side, and from Hercules by his mother's, (p. 35 ;) was instructed in medical science by his father, (p. 38 ;) travelled in different parts of the continent of Greece, (*ibid.;*) and died at Larissa, in Thessaly, (*ibid.*)[n] His two sons, Thessalus and Draco, and his son-in-law, Polybus, were also celebrated physicians, (pp. 36-7,) and are supposed to have been the authors of some of the works in the Hippocratic Collection. Such are the few and scanty facts that are told of the personal history of this celebrated man ; but, though we have not the means of writing a detailed biography, we possess in these few facts, and in the hints and allusions contained in various ancient authors, sufficient data to enable us to appreciate the part he played, and the place he held among his contemporaries. We find that he enjoyed their esteem as a practitioner, writer, and professor; that he conferred on the ancient and illustrious family to which he belonged, more honour than he derived from it; that he rendered the medical school of Cos, to which he was attached, superior to any which had preceded it or immediately followed it; and that soon after their publication, his works were studied and quoted by Plato. (p. 43.)

And now to proceed to the collection of works that go under the name of Hippocrates, of which we possess no fewer than sixty-four, as given in Kühn's edition.

"The first glance at these writings," says M. Littré (p. 44), "shows that they

[n] His age at the time of his death is uncertain, as it is stated by different authors at eighty-five, ninety, one hundred and four, and one hundred and nine years. (p. 38.)

form neither a whole, nor a [connected] body [of medicine], and that we should look in them in vain for the work of a man who had given his attention to all the different parts of medical science. The treatises not only do not refer to each other, but they even present the greatest dissimilarities. Some are complete in themselves; others are only collections of notes which follow one another without connexion, and which are sometimes hardly intelligible. Some are incomplete and mutilated; others form in the whole Collection particular series, which display the same thought and belong to the same author. In a word, however little we reflect on the context of these numerous writings, we are led at once to the conclusion that they are not the work of one and the same person. This remark has in all ages struck those critics who have given their attention to the works of Hippocrates, and even at the time when they commented on them in the Alexandrian school, they disputed about their authenticity. The manifest confusion which exists in the Collection, renders the intervention of criticism absolutely necessary; while, at the same time, the remote date at which these writings were composed, and the absence of direct testimony, render such an undertaking extremely difficult. And if the difficulties of the task were so great, and there was so much room for doubt, even in ancient times, what must be the case in the present day for us, who, since the time of the Alexandrian commentators and Galen, have experienced so many losses in every kind of literature? Many are the works which have had for their object the literary history of the Hippocratic Collection; many the eminent men that have given themselves up to the researches which this history demands; and yet, many are the questions which still remain undetermined, while the different and contradictory opinions held by critics on the authenticity or spuriousness of the same work, show that we have no fixed starting-point, nor any documents that are better than mere conjectures."

But it is not merely from internal evidence, (though this alone would be sufficiently convincing,) that we find that the Hippocratic Collection is not the work of that great man alone whose name it bears; for it so happens, that in two instances we find a passage that has appeared from very early times as forming part of this Collection, quoted as belonging to a different person. (p. 46, &c.)[o] Indeed, if we had nothing but internal evidence to guide us in our task of examining these writings in order to decide which really belong to Hippocrates, we should come to but few positive results, and therefore it is necessary to collect all the ancient testimonies that can still be found, (pp. 64-5;) in doing which, it will appear that the collection, as a whole, can be traced no higher than the period of the Alexandrian school in the third century before Christ, but that particular treatises are referred to by the contemporaries of Hippocrates and his immediate successors. (p. 65.)

We find that Hippocrates is mentioned, or referred to, by no less than ten persons anterior to the foundation of the Alexandrian school; the testimony of each of these individuals is given at length by M. Littré in his fourth chapter, (pp. 66-79,) and among them appear the names of Plato and Aristotle. The formation of the great library at Alexandria, in the third century before Christ, by which, in the reign of Ptolemy Philadelphus, that city succeeded Athens as the principal seat of ancient learning, is a remarkable era in literary history,[p] and especially in that of

[o] Aristotle quotes in his 'Historia Animalium,' (lib. iii, cap. 3,) a passage to be found in the treatise 'De Natura Hominis,' (tom. i, p. 364, ed. Kühn,) and attributes it to Polybus; and Galen quotes a fragment from Euryphon, (Comment. in Hippocr. 'de Morb. Vulgar., VI,' tom. xvii, pt. i, p. 888, ed. Kühn,) which forms part of the second book of the treatise, 'de Morbis,' (tom. ii, p. 284.)

[p] See Clinton's 'Fasti Hellen.,' vol. ii, pp. i, ii.

the Hippocratic Collection. (p. 80.) At this time the different treatises which bear the name of Hippocrates were diligently sought for and formed into a single collection, and about this time commences the series of commentators which has continued through a period of more than two thousand years to the present day. The first person who is known to have commented on any of the works of the Hippocratic Collection is Herophilus,[q] (p. 83 ;) the most ancient commentary still in existence is that by Apollonius Citiensis.[r] (p. 93.) Several other names are mentioned by M. Littré, (pp. 86-119,) but none of them deserve any special notice here, till we come to Galen, whose commentaries[s] are at the same time by far the most voluminous and the most valuable that remain, containing matter both historical, grammatical, and medical, and serving as a common storehouse of criticism, from which succeeding commentators, Greek, Latin, and Arabic, have freely borrowed. (pp. 119, &c.) The other ancient commentaries that remain are those of Palladius, Joannes Alexandrinus, Stephanus Atheniensis, Meletius, Theophilus Protospatharius, and Damascius,[t] besides a spurious work attributed to Oribasius,[u] and some Arabic commentaries that have never been published.

These critical works attest the general correctness of the text of certain treatises of the Hippocratic Collection ; but it is necessary to discover, if possible, what other works, besides those which we know to have been thus commented on, formed part of this collection in ancient times, and this inquiry is the subject of the sixth chapter. (pp. 133-53.) It appears that tables or lists (*πίνακες*) of the works that bore the name of Hippocrates were made some time before Galen ; the oldest of these, however, are now lost, (p. 133,) and the most ancient that still remains is that by Erotianus. (pp. 142-3.) M. Littré compares the different lists with each other, and with the Collection as we now possess it, but we cannot follow him through all the details of his work ; it may be sufficient to state, as the result, that our printed editions contain both more and less than was known to the ancients, (p. 148,) and that the treatises are differently arranged and divided from what used to be the case (pp. 149-50), and also bear, in some instances, different titles. (p. 150.) With respect to the data possessed by the ancients for judging of the genuineness of the different treatises, M. Littré considers that in three

[q] Professor Marx in his monograph on Herophilus, (pp. 13, 14,) supposes that he wrote *three* works on Hippocrates, (see Number XXIX of this Journal, p. 110 ;) this, however, is not quite certain, and M. Littré reckons only *one*. (pp. 83-5.)

[r] It is a short commentary on the treatise 'De Articulis,' and was published for the first time in the first volume of Dietz's 'Schol. in Hippocr. et Gal.' Regim. Pruss. 1834, pp. 1-50.

[s] We possess a Commentary by Galen on the works, 'De Natura Hominis ;' 'De Salubri Victus Ratione ;' 'De Ratione Victus in Morbis Acutis ;' 'Prænotiones ;' 'Prædictiones I. ;' 'Aphorismi ;' 'De Morbis Vulgaribus, I, II, III, VI ;' 'De Fracturis ;' 'De Articulis ;' 'De Officina Medici ;' and 'De Humoribus ;' with a glossary of difficult and obsolete words, and fragments on the 'De Aëre, Aquis, et Locis ;' and 'De Alimento.' We have lost his commentaries on the treatises 'De Ulceribus ;' 'De Capitis Vulneribus;' 'De Morbis ;' and 'De Affectionibus ;' together with a work on the Anatomical knowledge of Hippocrates, on his dialect, on the genuine writings, and on the marks (*χαρακτηρες,*) that are found in the third book 'De Morbis Vulgaribus.' (p. 119.)

[t] All of these are to be found in Dietz's 'Scholia' noticed above.

[u] This only exists (or, at least, has only been published,) in a Latin translation, of which we believe the last edition was that of Padua, 1658, 12mo.

points chiefly they had the advantage over the moderns. (p. 156.) The autograph MSS. were early lost (p. 155), nor was Galen ever able to discover any even as old as the date of the Alexandrian school, (p. 163;) but, in the first place, there seem to have existed certain traditions relating to the authors of the different works (p. 156, &c.) which were probably *essentially* true, though not correct in all their details, (p. 158;) secondly, the diligent examination of the medical writings belonging to the times immediately before and after Hippocrates, would enable us to fix two limits between which his genuine works must have been composed, (pp. 164-5); and thirdly, a work on the history of medicine, written by a pupil of Aristotle, and which must have been, from its antiquity, of very great value, is quoted by Galen (pp. 166-7), but is no longer extant. In these respects, then, as well as in several others, the modern critic meets with difficulties and checks which did not occur to his predecessors at the beginning of the Christian era; but then, on the other hand, a more searching, and (in some points,) a sounder spirit of criticism has grown up; perhaps, too, the very scantiness of our materials may make us more careful in making use of them; so that altogether, it will not appear presumptuous if we venture to differ in some of our results from what was said by Erotianus and Galen seventeen hundred years ago.

The earliest critic of modern times who undertook to determine which works in the Hippocratic Collection were genuine and which were spurious, was Lemos, who published a little work on this subject towards the end of the sixteenth century, (p. 169:)[v] as, however, he follows Galen implicitly, this attempt has not much critical value. (p. 170.) Next followed Mercuriali,[w] who divided the collection into four classes; viz. 1, works which bear the character of the style and doctrine of Hippocrates; 2, those which seem to be a mere collection of notes, intended for private use, and published after his death by his sons or son-in-law; 3, those which represent his doctrines with tolerable fidelity, but were written, not by himself, but by his pupils; and 4, those which do not belong either to Hippocrates or to his school. (p. 170.) Mercuriali argues chiefly from the style of the works, and attributes to Hippocrates certain characteristic peculiarities, (pp. 170-1,) as for instance, conciseness, obscurity, gravity, &c. &c., all of which is not only sufficiently vague, but also involves (as M. Littré observes,) a complete *petitio principii*, as, before describing the style of his author, he ought to have decided which were his genuine writings. (p. 171.)

It would be interesting and useful to draw up parallel lists of the works acknowledged as genuine by each of the ancient and modern critics, in order that the reader might see at one glance the amount of testimony in favour of each treatise; this, however, we are unable to do here, and must therefore be content with suggesting the plan to any one who takes an interest in the subject.

[v] It is entitled, 'Judicii Operum Magni Hippocratis Liber Unus,' Salmanticæ, 1584, (Choulant says, 1588,) fol.; Venet. 1592, 8vo; and Misenæ, 1835, 8vo, ed. J. G. Thierfelder. (See Choulant, 'Handbuch der Bücherkunde für die Aeltere Medicin,' Leipzig, 1841, 8vo.)

[w] Hieron. Mercurialis, 'Censura et Dispositio Operum Hippocratis,' Venet. 1583, (apparently *one* year, if not *five* years, *anterior* to the work by Lemos,) 4to; Basil. 1584, 8vo; Francof. 1585, 8vo; and in his edition of Hippocrates, 1588. (Choulant.)

Gruner has followed the same rules of criticism as Mercuriali (p. 176),[x] stating certain vague and unsatisfactory marks which he believes to be the characteristics of the genuine Hippocratic writings, and adding merely that those treatises in which a greater knowledge of anatomy is displayed, belong to a later period. (p. 177.)

Ackermann[y] (whom M. Littré considers as the surest guide to follow, (p. 196,) and who has fallen into the fewest errors,) accepts the rules laid down by Mercuriali and Gruner, but argues also from tradition and the agreement of the ancient critics. (p. 179.) Grimm[z] considers the testimony of antiquity as the most important guide, and after that, the internal evidence of style, &c., and Gruner's argument from the use of certain anatomical terms. (p. 179-80.)

All these rules (with the exception of that which depends on the testimony of the ancient critics,) were certainly unsatisfactory, as being in some cases vague, in others incorrect; though it must be allowed, that much progress had been made, as the field of criticism had been somewhat extended by each successive investigation since the time of Lemos and Mercuriali. The first person who made a really important addition to these rules was Sprengel,[a] who, by introducing the consideration of the different philosophical docrines that may be distinguished in the Hippocratic Collection, as a means of determining the order of priority of the different treatises, opened a new field of inquiry, which has since been pursued with distinguished success. (p. 181.)

M. Link[b] took for the basis of his criticism the medical and philosophical theories (p. 184,) of which he distinguishes six, and he accordingly divides the Collection into six classes, which he believes to be the work of at least six different authors. (p. 185.) The first class comprehends those treatises in which the theory of bile and phlegm appears (p. 185); in the second, may be distinguished that of the four humours (blood, black bile, yellow bile, and phlegm,) and that of the four elementary qualities, (hot, cold, dry, and moist,) (p. 188); in the third class he places only one treatise (*De Prisca Medicina*), in which the author argues against those who derived all diseases exclusively from the four elementary qualities, (pp. 189, 557;) the fourth theory considers fire to be the universal agent, (p. 193;) the fifth, air, (*ibid.;*) and the sixth makes mention of a morbific matter flowing down from the head, and extending over the whole body. (pp. 193-4.)

M. Littré considers Link's system of classification to be original and valuable (p. 194), but to be inadmissible in all its details, on account of three positive anachronisms with respect to medical doctrines. (p. 195.)

[x] C. G. Gruner, 'Censura Librorum Hippocrateorum, qua Veri a Falsis, Integri a Suppositis segregantur,' Vratislav., 1772, 8vo.

[y] He is the author of the article on Hippocrates in Harles's edition of Fabricii 'Biblioth. Græca,' (vol. ii, p. 535,) which has been reprinted with some additions by Kühn in his edition of Hippocrates.

[z] In his German translation of Hippocrates, Altenburg, 1781-92, 8vo; Glogau, 1837, 8vo.

[a] Kurt Sprengel, 'Apologie des Hippocrates und seiner Grundsätze, Leipzig, 1789, 1792. 8vo.

[b] H. F. Link, 'Ueber die Theorien in den Hippocratischen Schriften, nebst Bemerkungen über die Echtheit dieser Schriften,' in the 'Abhandlungen der K. Akademie der Wissenschaften in Berlin,' 1814-15.

This leads him to examine the light that may be thrown upon the authorship of the various works of the Hippocratic Collection, by the determination of the date of certain medical terms; and this question forms the subject of the ninth chapter. (pp. 200-41.)

Before, however, proceeding to this part of his introduction, it will be more proper to give some account of M. Petersen's little work, the title of which we have placed at the head of this article, and which, as it did not appear till after the publication of M. Littré's first volume, is carefully analysed and examined by him in the "Advertisement" to the second. M. Petersen is not a physician, but Professor of Classical Philology in the Gymnasium at Hamburg; and consequently he enters upon his subject with a greater store of classical learning than is commonly met with in the physicians of the present day, but at the same time labours under the disadvantage (which every non-professional person must necessarily feel,) of not being perfectly familiar with the subject-matter of the treatises before him. We hope, therefore, that he will not think us ungrateful or disrespectful if we venture to express our opinion that he would have done more service to the cause of ancient medical literature, if he had confined himself to questions of philology, and had endeavoured to determine the chronology of the different parts of the Hippocratic Collection, principally from a consideration of the varieties in style that may be traced in the several treatises composing it. This has never been attempted critically and systematically, (for the vague and indefinite remarks on the supposed style of the genuine works of Hippocrates, by Mercuriali and Gruner, do not by any means supply what is wanted;) and, as this seems to be almost the only question connected with the Hippocratic writings that has not by this time been thoroughly examined, we hope it will before long be taken up by some philologist, who will thus enable us to compare his conclusions with the results at which others have already arrived by a consideration of the various medical and philosophical theories contained in them.

M. Petersen takes as his basis the classification of M. Link, which, however, is modified in many of its details. He divides the works into nine classes, which he places in the following chronological order (pp. 13, 14): the first contains those treatises in which the flow of bile and phlegm is considered as the cause of disease; the second and third recognize respectively fire and air as the first principle of all things; in the fourth, bile and phlegm are spoken of as the primary humours of the human body; in the fifth, spirit (or pneuma, πνεῦμα,) and moisture are acknowledged as the first principles of generation; in the sixth, the elements of the body appear in opposition to each other; in the seventh, black bile, yellow bile, blood, and phlegm, appear as the primary humours of the human body; in the eighth, bile, water, phlegm, and blood, occupy the same place; and in the ninth, fire and water are supposed to be the first principles of all things.[c] To these he adds two other classes,

[c] We are almost afraid that our readers may think we owe them some apology and explanation for writing in such a strange and unintelligible language. We would, however, remind them that the purely theoretical parts of the ancient medical writers have never been considered the most valuable and interesting portions of their works; and we would gently hint that it is perfectly conceivable that a time may come when some of our modern theories and phraseology may be as much out of date, and as unintelligible to the ordinary medical practitioner, as those in the text now appear to us.

which could not enter into his chronological arrangement; one containing the works on surgery, the other those which consist of moral precepts, &c., and in which no medical doctrines can be traced.

M. Petersen promises to give to the world a second part of his work, in which he will examine each treatise of the Collection separately and more in detail, (pp. 6, 7;) perhaps, therefore, we may be thought premature in noticing his conclusions before we have seen the whole of the evidence in support of them. We shall, indeed, be anxious to see the second part of his work; but in the mean time, we confess that we think it will be difficult to convince us that M. Petersen has not committed in the classification of the different writings of the Hippocratic Collection, some few oversights, of which two striking instances are noticed by M. Littré. (tome ii, pp. 32-3.) We think, also, that he is wrong in some parts of the personal history of Hippocrates, but these we have not room here to specify.

We must now return to our analysis of M. Littré's introduction to his first volume, in which, after having finished his account of the modern classifications of the Hippocratic writings, he proceeds (chap. ix, p. 200, &c.,) to discuss several points of medical chronology, preparatory to laying before the reader his own opinion on the authorship of each treatise. This is to ourselves a very interesting chapter, but, as an analysis of it might not prove equally so to the greater part of our readers, we must content ourselves with stating merely the results at which M. Littré arrives. It appears, therefore, (p. 241,) that in the Hippocratic writings the science of anatomy is very imperfect, except on certain points connected with surgery; the arteries are supposed to be full of air, or are called, together with another set of canals, by the common name of *veins*, (p. 214;) the relation of the blood-vessels with the heart is considered as of little importance, (p. 241;) mention is made of the pulse, but its application and use in medicine are quite unknown, (p. 230;) the word *μῦς*, *muscle*, is *not* used first by the anatomists of Alexandria, and therefore no argument can be founded on the fact of its being found in this or that treatise, (pp. 230-1;) the nerves are mentioned in a vague and obscure manner under the name of *τόνοι*, but their functions and different relations are evidently not known, (p. 234;) and lastly, M. Littré is inclined to think that the human body had been examined by actual dissection with more or less exactness before the time of the Alexandrian physicians. (p. 238.)

In the next chapter (p. 242, &c.) M. Littré proceeds to examine with a considerable degree of minuteness, the connexion between certain works in the Hippocratic Collection, showing that, in many instances, not only the same sentiments, but even the same words, are to be found in two or more of them, and that some treatises are entirely made up of fragments taken from other parts of the Collection, and put together without connexion or order.

With respect to the manner in which the Hippocratic Collection was formed and given to the world, (which forms the subject of the next chapter, p. 262, &c.,) M. Littré first points out eight principal and characteristic circumstances which an attentive examination may discover, (p. 263:) 1st, this Collection does not exist in an authentic form earlier than the date of Herophilus and his pupils in the third century before

Christ; 2d, it contains passages which certainly do not belong to Hippocrates himself, 3d, collections of notes, &c., which would never have been published by their author in their present form, 4th, and also compilations which are either abridged, or copied word for word, from other works which still form part of the Collection; 5th, as the different treatises do not all belong to the same author, so neither were they all composed at the same time, some being much more modern than others; 6th, we find in different parts of the Collection itself, mention made of numerous treatises written by the followers of Hippocrates, which are now lost, and which were no longer in existence when the Collection was first published; 7th, the most ancient critics did not know for certain to whom the several works forming the Collection belonged; 8th, with the exception of a small number, which all of them, for one reason or another, agreed in attributing to Hippocrates himself. Upon these eight propositions (which he expands and illustrates,) M. Littré founds his hypothesis respecting the occasion and manner in which the Hippocratic Collection was first published. He imagines that the genuine works of Hippocrates, together with the rest of his library, continued in the possession of his descendants, the Asclepiadæ; that this family became extinct about the beginning of the third century before Christ, (pp. 285-6,) upon which these works, as well as those which had been composed and added to the library after his death, fell into the hands of persons who knew neither their value nor their origin, except that they came from the followers of Hippocrates, (p. 282;) and that in this state they were placed shortly afterwards in the newly formed library at Alexandria. Their collection into their present form, and the confusion that exists among them, he illustrates by the somewhat similar history of the works of Aristotle, (p. 282, &c.,) which indeed may perhaps have suggested to him the details of his hypothesis. But, however this may be, his account is (like most hypotheses) sufficiently ingenious, and upon the whole, though not in all its details, sufficiently probable to satisfy us in a matter where no certainty is to be obtained.

The twelfth chapter contains an examination of each treatise in the Hippocratic Collection separately, with a view to determine its date and author. (pp. 292-439.) Of this chapter we cannot pretend to give anything like an analysis, as great part of it would hardly admit of abridgment, and it is besides calculated rather for being consulted respecting any particular work, than for being read through. We have ourselves, however, read every page, and may therefore venture to say that, though we do not agree with the writer in all his conclusions, yet upon the whole, we consider it by far the best classification of the Hippocratic writings that has yet been given to the world. We must content ourselves with giving M. Littré's results, without touching upon his arguments in support of them; but before doing this, we may mention the four rules which he has followed in his classification. (p. 292.) The first and most important argument in favour of the genuineness of any of the Hippocratic writings, is derived from the direct testimony of authors who lived before the formation of the library at Alexandria; the second depends on the consent of the ancient critics, who, in many cases (as we have seen in the seventh chapter,) possessed documents to guide their opinion, which no longer exist; the third rule consists in the application of certain points of medical history, by which the date of a treatise is determined; and the fourth results from the consideration of doctrines, the difference of style, &c.

The Hippocratic Collection consists of more than sixty works,[d] and these are classed in eleven divisions by M. Littré, according to the following system, (p. 436:) he first endeavoured to find a fixed point from which to start in his inquiries, and accordingly began by determining which are the genuine works of Hippocrates;[e] when this was done, it appeared, from comparing his genuine works with the rest of the Collection, that some of the treatises were earlier than his time,[f] (inasmuch as he had made use of them in some of his writings,) and others later. Hence arise three simple and distinct classes, of which the last is subdivided into several others. Of the writings later than the time of Hippocrates, M. Littré attributes two to Polybus,[g] and one, (though doubtfully, and as it seems to us, without much reason,) to Leophanes.[h] Several seem to form a series, and to have been written by the same individual, who is, however, entirely unknown.[i] There still remain a great number of works to be disposed of, several of which bear evident traces of belonging to the school of Cos, and of being pretty nearly contemporary with Hippocrates;[k] while others appear, either from internal or external evidence, to have been written about the time of Aristotle and Praxagoras;[l] and others again consist merely of notes, extracts, &c., which have evidently not been prepared for publication, but which have formed part of the Hippocratic Collection from the earliest times.[m] There remains nothing more except a certain number of smaller works, which are classed together: 1st, as having this at least in common, viz., that they are not mentioned even by name by any of the ancient writers whose works are still extant;[n]

[d] Like the question as to the number of the bones in the human body, it is not possible to say exactly *how many* treatises the Collection contains, or at least not without carefully specifying, not only which works are meant to be included, but also whether some of these are reckoned as one single treatise, or as several.

[e] This forms his first class, in which he comprehends the works, 'De Veteri Medicina;' 'Prænotiones,' (or 'Prognosticon;)' 'Aphorismi;' 'De Morbis Popularibus,' (or 'Epidemiorum,') lib. I and III; 'De Ratione Victus in Morbis Acutis,' (or 'De Diaeta Acutorum;') 'De Aëre, Aquis, et Locis;' 'De Articulis;' 'De Fracturis;' 'Vectiarius,' (or 'Mochlicus;') 'De Capitis Vulneribus;' 'Jusjurandum;' and 'Lex.'

[f] Class III, containing the 'Prænotiones Coacæ,' and the first book of 'Prædictiones,' (or 'Prorrhetica.')

[g] Class II, containing 'De Natura Hominis,' and 'De Salubri Victus Ratione.'

[h] Class VII, containing 'De Superfœtatione.'

[i] Class VI, containing 'De Genitura;' 'De Natura Pueri;' 'De Morbis,' lib. IV; 'De Mulierum Morbis;' 'De Virginum Morbis;' and 'De Sterilibus.'

[k] Class IV, containing 'De Ulceribus;' 'De Fistulis;' 'De Hæmorrhoidibus;' 'De Morbo Sacro;' 'De Flatibus;' 'De Locis in Homine;' 'De Arte;' 'De Victus Ratione,' (or 'De Diæta;') 'De Insomniis;' 'De Affectionibus;' 'De Internis Affectionibus;' 'De Morbis,' lib. I, II, and III; 'De Septimestri Partu;' and 'De Octimestri Partu.'

[l] Class VIII, containing 'De Corde;' 'De Alimento;' 'De Carnibus;' 'De Septimanis;' 'Prædictiones,' (or 'Prorrhetica,') lib. II; 'De Glandulis;' and a fragment of 'De Natura Ossium,' called 'De Venis.'

[m] Class V, containing 'De Morbis Popularibus,' (or 'Epidemiorum,') lib. II, IV, V, VI, and VII; 'De Officina Medici;' 'De Humoribus;' and 'De Usu Liquidorum.'

[n] Class IX, containing 'De Medico;' 'De Decenti Habitu;' 'Præceptiones;' 'De Resectione Corporum,' (or 'De Anatomia;') 'De Dentitione;' 'De Visu;' 'De Natura Muliebri;' 'De Resectione Fœtus;' the eighth section of the 'Aphorisms;' 'De Natura Ossium;' 'De Judicationibus,' (or 'De Crisibus;') 'De Diebus Judicatoriis,' (or 'De Diebus Criticis;') and 'De Remediis Purgantibus.'

2d, some others which are manifestly apocryphal;[o] and 3d, the names of three which no longer exist.[p]

Such is M. Littré's classification of the Hippocratic writings, which we think certainly superior to any that has preceded it, though we do not so entirely agree with all its details as to believe it impossible to be improved. The author next proceeds (in the thirteenth chapter, p. 440, &c.,) to give a summary of the medical doctrines of Hippocrates; from which, however, he excludes anatomy and physiology, as being too little understood at that time for him to have had any but vague and imperfect ideas on the subject. (p. 441.) It will at once be seen that, before analysing a man's opinions as they appear in his writings, it is a most indispensable preliminary step to determine which are his genuine works, and which are spurious. This plain rule, however, has not always been very strictly attended to in the case of Hippocrates, either by ancient or modern critics, and the consequence is, that, not only in his intellectual, but also in his moral character, he has received both praise and blame, to which he is not justly entitled.[q] From observing this distinction, M. Littré's analysis of the medical opinions of Hippocrates, though short, is more satisfactory than any with which we are acquainted. Hippocrates divides the causes of disease into two principal classes; the one comprehending the influence of seasons, climates, water, situation, &c., as developed in the treatise *De Aëre, Aquis, et Locis;* and the other consisting of more personal and private causes, such as result from the particular kind and amount of food and exercise in which each separate individual indulges himself, (p. 442,) and which are particularly dwelt upon in his little work *De Prisca Medicina.* The modifications of the atmosphere dependent on different seasons and climates is a subject which was successfully treated by Hippocrates, and which is still far from exhausted by all the researches of modern science. He considered that while heat and cold, moisture and dryness, succeeded one another throughout the year, the human body underwent certain analogous changes, which influenced the diseases of the period, (*ibid.*;) and on this basis was founded the doctrine of pathological constitutions, corresponding to particular conditions of the atmosphere, so that whenever the year or the season exhibited a special character in which such or such a temperature prevailed, those persons who were exposed to its influence were affected by a series of disorders, all bearing the same stamp. (*ibid.*) How plainly the same idea runs through the great work of our English

[o] Class XI, containing the 'Epistolæ;' 'De Insania;' 'De Veratri Usu;' 'Salubre Consilium ad Regem Demetrium;' 'Atheniensium Senatus-consultum;' 'Oratio ad Aram;' and 'Thessali Legati Oratio.'

[p] Class X, containing 'De Mortiferis Vulneribus;' 'De Telis et Vulneribus;' the first book 'De Morbis,' called 'the Less,' (ὁ σμικρότερος).

[q] We may mention two instances of this, both taken from ancient authors. Celsus in a well-known passage, (lib. viii, cap. 4,) extols his magnanimity in confessing that he had been once deceived by the sutures of the cranium, and had overlooked a fracture in consequence; the passage referred to, however, is to be found in the fifth book, 'De Morbis Popularibus,' (tom. iii, p. 561,) which is universally considered to be spurious. On the other hand he is commonly accused of having produced abortion, on account of a passage in a treatise which he probably never wrote, ('De Natura Pueri,' tom. i, p. 385,) and which one of his ancient commentators, (Joannes Alexandrinus in Dietz's 'Scholia in Hippocr. et Gal.' tom. ii, p. 216,) tries in vain to defend and to reconcile with the Hippocratic 'Oath.'

Hippocrates, need hardly be pointed out. The belief in the influence which different climates exercise on the human frame follows naturally from the theory just mentioned; for in fact, a *climate* may be considered as nothing more than a *permanent season,* whose effects may be expected to be more powerful inasmuch as the cause is ever at work upon mankind. (*ibid.*) Accordingly, Hippocrates attributes to climate both the conformation of the body, and the disposition of the mind—indeed, almost everything; and if the Greeks are found to be hardy freemen, and the Asiatics effeminate slaves, he accounts for the difference of their character by that of the climates in which they live. (p. 443.) The analogy between the seasons of the natural year, and the different periods of the human life, is in some respects no less medically, than poetically, correct; and it was more easy for Hippocrates to dwell on this parallel, as it would agree with his theory respecting innate heat, which he supposed to be at its maximum in the human body during infancy, and, while gradually diminishing during life, to undergo certain changes corresponding in a manner to those of the earth during its annual revolution round the sun. (p. 443.)

With respect to the second class of causes producing disease, he attributed all sorts of disorders to a vicious system of diet, which, whether excessive or defective, he considered to be equally injurious; and in the same way he supposed that, when bodily exercise was either too much indulged in, or entirely neglected, the health was equally likely to suffer, though by a different form of disease. (*ibid.*) Into all the minutiæ of the humoral pathology, which kept its ground in Europe as the prevailing doctrine of all sects for more than twenty centuries, we need not now enter. It will be sufficient to remind our readers that the four fluids or humours of the body (blood, phlegm, yellow bile, and black bile,) were supposed to be the primary seat of disease; that health was the result of the due combination (or *crasis*) of these, and that, when this crasis was disturbed, disease was the consequence, (p. 446;) that, in the course of a disorder that was proceeding favorably, these humours underwent a certain change in quality (or *coction*), which was the sign of returning health, (p. 447, &c.,) as preparing the way for the expulsion of the morbid matter, or *crisis*, (p. 450;) and that these crises had a tendency to occur at certain stated periods, which were hence called *critical days.* (p. 451.) From the general consideration of the causes of disease, and from the several parts of the humoral theory, resulted (what was called in the time of Hippocrates,) *prognosis*, (*ibid.;*) a word which related not only (as it is now used by us,) to the future, but also to the past and present condition of the patient, (p. 452,) and which, in fact, comprehended no less than the whole of the *science* of medicine, (properly so called,) without which there remained nothing but a blind empiricism. (p. 454.)[r] The account given by M. Littré of the medical practice of Hippocrates, is extremely meagre, nor do we quite understand why he reckons the treatise *De Ratione Victus in Morbis Acutis* as a work on therapeutics, (indeed, as the

[r] For a further explanation and development of this subject, our readers must be referred either to the 'Prognosticon' of Hippocrates himself, or to M. Littré's able introduction to that treatise in his second volume.

*only* work by Hippocrates that we possess on this subject, p. 461,) when he begins the argument prefixed to it (vol. ii, p. 192,) by saying that its object is not to explain the treatment of acute diseases in general, but only to touch on one particular point of practice, viz., the diet of the patients. We cannot, however, attempt to supply the deficiency here, farther than by saying that the medical practice of Hippocrates appears to have been very much what might have been expected by any person acquainted with his system of pathology; in short, it consisted chiefly in watching the operations of Nature, and promoting the critical evacuations mentioned above—a plan of proceeding sufficiently cautious and inert, but which, (as Dr. Bostock remarks,) considering the state of medical knowledge at that time, must be admitted to be much more salutary than the opposite extreme.

With regard to the medical character and style of Hippocrates, (which forms the subject of the last chapter, pp. 465-78,) although he says little or nothing expressly about himself, and exhibits in this respect, a striking contrast to the interesting loquacity and egotism of Galen, yet it is impossible to avoid drawing certain conclusions from the characteristic passages scattered through the pages of his writings. He was evidently a person who had not only had great experience, but who also knew how to turn it to the best account; and the great number of moral reflections and apophthegms that we meet with in his writings (several of which have acquired a sort of proverbial notoriety,) show him to have been a profound thinker. He loved his profession, and was jealous for its honour, (pp. 467-8,) while for every species of quackery and charlatanism, he had the utmost aversion and contempt. (pp. 469-70.) He appears also to have felt the moral obligations and responsibility of his profession, and often tries to impress upon his readers the duties of care, attention, and kindness towards the sick, saying that a physician's first and chief consideration ought to be the restoring his patient to health. (p. 467.) His style is (as every one knows,) so concise as to be sometimes extremely obscure; though this charge, which is as old as the time of Galen, is often brought too indiscriminately against all the works of the Hippocratic Collection, whereas it applies, in fact, especially only to certain treatises which seem to be merely a collection of notes. (p. 473.)[s] In those writings, which are universally allowed to be genuine, we do not find this excessive brevity, though even these are in general by no means easy.

M. Littré concludes his Introduction with some excellent observations on the study of the ancient medical writers, (p. 475, &c.,) which we cannot well abridge, and which we have not room to translate, but which seem to us to suit exactly two classes of persons, viz., those who imagine on the one hand, that an ancient treatise on medicine can by means of certain explanatory and corrective notes be made to supersede the use of a modern work on the same subject; and, on the other hand, that much more numerous class (including some celebrated names, both of the last and the present generation,)[t] who find it easy and convenient to

[s] Such as 'De Humoribus, De Alimento, De Officina Medici,' &c.
[t] An instance of this is mentioned in our last April Number, p. 439.

sneer at learning, which they do not themselves possess, and therefore, from sheer ignorance, believe their forefathers to have been only egregious blockheads.

The Introduction is followed by an Appendix, treating of the Ionic dialect as it appears in the works of Hippocrates, (pp. 479-502,) of the editions published by the ancients, (pp. 502-11,) of the MSS. in the libraries at Paris, (pp. 511-40,) and of the modern editions and translations of the whole Collection. (pp. 540-54.) This, however, as being probably uninteresting to the greater part of our readers, we must omit, though we should be glad to see the question of the dialect examined at length by some scholar competent to the task. The remainder of the volume (pp. 557-637) is taken up with one single treatise, (*De Antiqua Medicina,*) to which is prefixed an argument, containing all the information necessary for the due understanding of the work. M. Littré's second volume begins with an Advertisement, (pp. v-xlviii,) in which he examines some works connected with the Hippocratic Collection, which were not published, or which he had not seen, at the time of the appearance of his first volume: one of these is M. Petersen's Dissertation, of which we have already given some account. The treatises contained in the second volume are, *De Aëre, Aquis et Locis, Prænotiones, De Ratione Victus in Morbis Acutis*, and the first book *De Morbis Popularibus*, all edited and translated on the same plan, and with the same care. The third volume also contains an Advertisement devoted to the examination of some works which the editor had not before seen, (pp. v-xlii,) and four of the works of Hippocrates, viz., the third book *De Morbis Popularibus, De Capitis Vulneribus, De Officina Medici*, and *De Fracturis*. The fourth volume we have seen advertised, but, though we have inquired for it more than once, we have not yet been able to obtain it; we therefore thought it due both to M. Littré and to ourselves, to delay the notice of his work no longer. Our general opinion of it has been already expressed: that it will supersede the necessity of a new edition for the next fifty or even twenty years, we are far from venturing to affirm; but that it is (as far as it goes,) the standard critical edition, that it does more towards settling the text than any that has preceded it, and that its use is indispensable to every physician, critic, and philologist, who wishes to study in detail the works of the Hippocratic Collection, we can assert without much fear of contradiction.

